# Hospital and Institutional News

**Published:** 1916-05-06

**Authors:** 


### HOSPITAL AND INSTITUTIONAL NEWS.

### DECORATIONS FOR CONSULTANTS WITH THE ARMY.

The latest *Gazette* of War Honours (May 2) notifies the bestowal of many decorations upon distinguished physicians and surgeons who are serving with the Army and in consultative capacities. Thus the C.B. is conferred upon Temporary Colonels William Hunter, M.D. (physician to Charing Cross Hospital, and for many years dean of the school there); C. A. Ballance, F.R.C.S. (chief surgeon to the Metropolitan Police, surgeon to St. Thomas's, and consulting surgeon to the National Hospital, Queen Square); A. W. Mayo Robson, F.R.C.S. (the well-known Leeds surgeon, who gave up the well-earned leisure of retirement from practice in order to serve his country); Y. W. Low, F.R.C.S. (surgeon to St. Mary's, and an old African campaigner); J. Purves Stewart, M.D. (physician to Westminster Hospital, and specialist in nervous diseases); Charles J. Symonds, F.R.C.S. (surgeon to Guy's Hospital); W. Thorburn, F.R.C.S. (an eminent Manchester surgeon); C. S. Ryan, M.B. (a Melbourne surgeon of much military experience); F. D. Bird, F.R.C.S.; and Sir Victor Horsley (the University College and National Hospital surgeon). It will be seen that the majority of these are London consultants, but that the provinces and the Colonies are not overlooked.

### THE ORDER OF ST. MICHAEL AND ST. GEORGE.

Then follow several appointments to the Most Distinguished Order of St. Michael and St. George. Surgeon-General Babtie, V.C., who was lately appointed to help Sir A. Keogh at the War Office, is promoted from Companion to Knight Commander of that Order. New Companions are Temporary Colonels A. H. Tubby, F.R.C.S. (Westminster Hospital, and well known as an orthopædic surgeon); A. E. Garrod, M.D. (physician to St. Bartholomew's and other hospitals); F. M. Sandwith, M.D. (a specialist on tropical, especially Egyptian, diseases); and W. H. Willcox, M.D. (St. Mary's Hospital, and scientific adviser to the Home Office). The same decoration is also bestowed upon a Territorial officer, Temporary Lieut.-Colonel C. H. Lindsay, M.D.; and upon three Canadian medical officers—Lieut.-Colonels F. Etherington and S. H. McKee, and Major E. G. Davis. Finally, the D.S.O. is conferred upon Lieut.-Colonel E. V. Gostling, R.A.M.C., T.F.; and upon Major J. G. Bell, M.B., R.A.M.C. The Military Cross is awarded to Temporary Captain W. N. Rushworth, M.B., R.A.M.C.

### "AND SOME THERE BE WHICH HAVE NO MEMORIAL."

In performance there has been no failure in the personal heroism of the race. Some of the tales are told and some of the actors are recognised. But these are only examples of a great host who have no mention in this world's pages. There has been again and again the swift instinct of unselfish and heroic action, the uncalculated daring, and the endurance even unto death. The gloomy prophets of to-day do not see the meanings of these things because they isolate them as exceptional events, whereas the message of all the fighting speaks ever of a high standard both of courage and chivalry. The melancholy strains of race degeneration which were heard in the past ring false in the light of to-day's achievements. And all the heroism is not in the trenches. There are countless homes throughout the country where the sharpest anxiety is a daily guest and not a few in which pain and loss have taken an abiding possession. Yet the suffering is borne silently and with the conviction—sometimes almost triumphant, sometimes with a vague longing—that "good will be the final goal of all." Take the story in its completeness and our people have not been unequal to the event nor unworthy of the traditions which they carry. It is a great thing to say, and yet it is the most confident of the truths. In such thoughts there is consolation and confidence, and even seeming failure is redeemed. Doubtless there are other faiths and hopes and words of comfort which are hardly for discussion here. Our concern is with less exalted messages, which, though they make no pretence to a complete answer to the riddle, speak not uncertainly in tones of good report. Despair of the world and of man is not the teaching of the war. It speaks rather of "exultations, agonies, and love, and man's unconquerable mind."

### SHAKESPEARE ON MEDICINE.

Just as nearly every profession, including even that of the soldier, has tried to claim Shakespeare, so it has been the delight of each to gather together every allusion in his writings which displays, or may be twisted to suggest, a profound knowledge on his part of its technicalities. Shakespeare literature includes a respectable number of books on his relation to medicine, and the Medical Society of London celebrated his tercentenary by inviting Sir St. Clair Thomson, M.D., to deliver the annual Oration on May 1, when he chose as his subject "Shakespeare and Medicine." With the subject of the Oration we propose to deal, in the space that it demands, next week. For the present we confine ourselves to acknowledging the mass of quotations, bearing on the subject, which the writer has compiled. As the Medical Society of London possesses in its library the commonplace book of the Rev. John Ward, Vicar of Stratford-on-Avon, in which is recorded "the only authentic record" of Shakespeare's death, describing his last drinking bout with Ben Jonson and Drayton, there is a personal association between the Society and Shakespeare which creates a bond between them. The Rev. John Ward makes three references to the poet: the one referred to; one to the money he made as a playwright; and the third to the marriage of one of his two daughters to "M. Hall, the physician." The commonplace book covers the years from 1648-1679. After illustrating Shakespeare's medical knowledge by abundant quotations, Sir St. Clair concludes thus: "The science of medicine progresses, but human nature remains the same. Shakespeare's plays will be read by physicians when every medical treatise of the present year will have been completely eclipsed." Yes; but doctors, like other men, will read him because he was a great poet, with the poet's capacity for absorbing knowledge and magical powers of language, not because of the medical, legal, typographical, or military tit-bits in his works.

### THE R.A.M.C. AND CIVILIAN SICK.

A special correspondent of the *Times* has recently remarked upon the extent of the services rendered by the R.A.M.C. to the civilian sick in those parts of France and Belgium which are occupied by our troops. It is one of the distinguishing characteristics of the peasantry in the regions of our battle-line that they will not willingly be divorced from their cottages and fields in the fire zone, even quite near the trenches. The result is that occasional casualties occur, and there is no one to attend to such wounded men, women, or children, or to any sick there may be, except the doctors of the R.A.M.C., whose services are frequently asked for and never refused. Another aspect of medico-military work which brings our officers in contact with the civil population is the prevention of epidemics. Hygiene and sanitation are practically unknown among the Belgians and the French in country districts; and the tracking down of the source of epidemics often discloses sanitary defects which have to be remedied for the sake both of the inhabitants and of our troops. Yet again, another branch of practice which our officers are constantly called upon for is midwifery: the local doctors are all called up for Army service, and though many of the country midwives remain, they find difficulty in getting from case to case because of the military regulations forbidding night travelling on roads in the military area. It is gratifying to see that the work of the R.A.M.C. in giving free attendance to civilian Allies is being recognised in lay newspapers.

### A SCOTTISH HERO.

The Londonderry wing of the Ulster Volunteer Force Hospital, Belfast, was the scene of an interesting ceremony on April 19, when Lance-Sergeant S. Rogers, of the 4th (Ross Highland) Battalion Seaforth Highlanders, T.F., was presented with the Distinguished Conduct Medal for, to quote official language, "conspicuous bravery, when he carried a wounded man over open ground and under heavy fire into safety, returning at once to his place in the firing line. He also assisted to collect wounded until dawn, and again the following night." The ceremony marked a red-letter day for the hospital, and the presence of Brigadier-General G. Hackett Pain, C.B., who made the presentation; Lieut.-General Sir George Richardson, K.C.B., chairman of the U.V.F. Hospital; the Lord Mayor of Belfast, and many others, including the nurses and convalescent soldiers, was evidence of the importance with which the occasion was regarded. Sergeant Rogers has been seven months in the wards, and it is reported that he is well on the road to complete recovery. That he is respected and admired by his fellow-patients is shown by the presentation on the same occasion of an illustrated testimonial signed by them, to which the committee of the hospital added a little present in the shape of some Treasury notes.

### A HOSPITAL PROJECT TO BE DEFEATED.

In a circular which has lately been sent broadcast to the medical profession, Mr. C. J. Heath announces that he is about to open a new hospital and clinic for ear disease in London. So far details of the proposal have not reached us; but we note that Mr. Heath is convinced that London needs five times as many beds and five times as many surgeons as are now available for the treatment of ear disease, especially suppurating otitis media. We cannot agree with this view, and the proposal is one we hope the public will refuse to pay for.

### OLD HOSPITAL LIFE IN NORWICH.

Mr. E. B. Southwell, in presiding over the recent annual meeting of the Norfolk and Norwich Hospital, brought to light some interesting points in the hospital's history, enshrined, as he explained, in "ancient correspondence, old newspaper cuttings in private ownership, and in the extracts of the written transactions of the hospital which were published by the late Sir Peter Eade." Mr. Southwell, in recalling the foundation of the hospital in 1772, gave the following, among other, curious details. The first beds purchased were four-posters hung with curtains. Feather bolsters, but no pillows, were allowed. (The horror of flock is a comparatively modern improvement.) We give this merely as a sample of the interest which painstaking research can reveal; but the whole account makes a past phase of hospital administration live again so well that we quote the entire passage elsewhere in this issue. Mr. Southwell deserves thanks for the interest of his speech, and that he was able to make it should encourage others to follow his example, and that too of the late Sir Peter Eade, in compiling details of every hospital's history. This is a mine which has been far too little worked in the past.

### A HOSPITAL WITHOUT A TREASURER.

The Walsall and District Hospital has been in the somewhat peculiar position of being without a treasurer owing to the difficulty of finding a suitable candidate with enough time to spare to devote to this post. The difficulty has now been overcome, however, by the appointment of Mr. R. E. Ledbury, who has long been connected with the institution, and has interested himself particularly in the workpeople's subscriptions. In accepting the position Mr. Ledbury remarked that since he had joined the executive committee the working-men's subscriptions had increased, and he and they were bent on raising this source of income to £2,000. For a hospital to be without a treasurer for any length of time is so obviously undesirable that Mr. F. J. Cotterell, the chairman, must be relieved to see the vacancy filled.

### DISAPPOINTED BOGNOR.

THE politicians lately have been blamed for the adoption of a halting policy, and for delays and similar signs of weakness; but the military authorities, so far as hospital accommodation is concerned, have also changed their minds fairly often. This has been the case in connection with the various schemes for the establishment of hospitals on the South Coast, which eventually have come to nothing, much to the disappointment of the seaside towns, which hoped to give and receive benefits through the presence of the wounded. The clerk to the Bognor Urban District Council, who wrote to Brigadier-General Baker Brown, Eastern Command, asking whether the War Office had abandoned their scheme for establishing a war hospital at Bognor, has received the following reply: I am sorry to see the War Office have again decided not to construct a hospital at Bognor. I regret this, and I feel sure that the facilities of fine air, good beach, and general comfort which the town affords would have been of benefit to our wounded soldiers. The word "again" in this note is sufficient to account for the local disappointment which has been felt.

### A CLEVER ESCAPE FROM AN ASYLUM.

A PATIENT recently escaped from Claybury Asylum under peculiar circumstances. She obtained access to a nurse's room, put on the nurse's private clothes, and walked out of the nurse's entrance and disappeared. After the statutory interval of fourteen days she was discharged "by operation of law." How she obtained the pass which is usually demanded of a nurse going out on leave is not stated. She was well known in several London hospitals as the actor of imitation epileptic fits, and during her stay in the asylum had suffered from attacks that were epileptic in appearance but were diagnosed as hysterical. What symptoms of madness she presented are not stated, and if these attacks constituted the whole malady it is difficult to understand how she came to be certified. As she wrote subsequently to say that she was in a comfortable situation and earning her livelihood, it appears that whatever symptoms of insanity she presented during her stay in the asylum, her malady did not interfere with the earning of her living; and still the wonder grows to understand, first, why she was ever sent to the asylum, and, second, why she was kept there. We dare say further knowledge of the case would clear up the mystery, but up to the present a mystery it is.

### THE BOY AND HIS FUTURE.

IN Devonshire last week a boy of fifteen was out with his dog, when the animal was caught in a gin. The boy was so infuriated that he ran amok. He smashed fences, he unhung gates, and threw one across the railway line, and finally he detached the coping-stone of a bridge on to the line, where it injured a passing engine, and narrowly missed causing a serious accident with loss of life. The boy was sent for four years to a reformatory, and the sentence was no doubt deserved and appropriate. There he will be under discipline, and will be trained to control himself. The question naturally arises what the prospect is for the boy's future. Will he grow up into a law-abiding and moral man, or is he a potential criminal, and sure to develop into an actual criminal? Well, in the first place, everyone is a potential criminal; and whether he becomes an actual criminal depends on many things —training, temptation, opportunity, and so forth. Lombroso and his school, if there is anyone of his school left, would say, of course, that this boy is a born criminal. They would find that his head is oxycephalic, or brachycephalic, his nose too long or too short, his ears of the wrong form, and would stamp him as a hopeless criminal. Fortunately, the school of Lombroso is almost as dead as he is himself. Experience shows that the very great majority of children who show criminal propensities at an early age grow up into moral and law-abiding men and women; and the particulars of this boy's crime do not lead us to regard him unfavourably. Undoubtedly his action was very dangerous and abominable, and, what is more to the purpose, he was quite old enough to know how dangerous and abominable it was; but the root of the action was not at all bad. It was not selfish or self-indulgent. On the contrary, it was generous. It arose out of sympathy for the cruelty inflicted on his dog. The boy is, no doubt, passionate and wanting in self-control; but he is not a bad boy. He is not selfish nor unfeeling, and with discipline and judicious management he will do all right.

### DEATH UNDER AN ANÆSTHETIC AT THE ANTI-VIVISECTION HOSPITAL.

THE usual verdict of "Death from misadventure" was returned at an inquest on a stoker, aged thirty-five years, who died at the Anti-Vivisection Hospital, Battersea, whilst undergoing an operation for the removal of an abscess formed in the neck. Dr. R. Frazer, the house surgeon, administered chloroform to the deceased, whose heart was normal, and who had been prepared for the anæsthetic. When Dr. Frazer was taking the mask off after the operation he saw the deceased change colour and stop breathing. Strychnine was injected, oxygen was given, and artificial respiration was administered, but without avail. Dr. W. L. Maccormac, medical superintendent of St. James's Infirmary, Wandsworth, who made a *post-mortem* examination, said that the neck was hard and swollen from acute inflammation of the cellular partition. The right side of the heart was dilated, and it was flaccid. There was considerable swelling of the glottis from dropsy or œdema, and the lungs and other organs were gorged with blood. Death was due to asphyxia, caused by œdema of the glottis due to the inflammation and the anæsthetic. The Coroner remarked that everything possible seemed to have been done after deceased ceased breathing. The anæsthetic used was the right one, it was properly administered, and the operation was properly conducted. Upon which we have merely to observe that if there is any one kind of case in which the services of a specially skilled anæsthetist are more required than in any other, it is in cases of deep suppuration in the neck. The dangers of anæsthesia in these cases are so well known that no man of the standing of a hospital resident should be entrusted with it unless in grave emergency. Further, since œdema of the glottis is the imminent danger in these cases, preparations for instant tracheotomy, and speedy resort to that operation in case of need, are amongst the elementary necessities of the operation.

### AN APPRECIATED RETURN TO DUTY.

GENERAL satisfaction has been expressed in Wandsworth at the return to duty of Dr. W. L. Maccormac, Medical Superintendent of St. James's Infirmary, after a long illness. The Wandsworth Board of Guardians have decided to send him a letter congratulating him on his restoration to health. Canon Curtis, the chairman of the board, voiced the unanimous opinion of his colleagues when he said they were all pleased to know that the doctor was once again with them. He had undergone a severe illness, and it was a wonder that his life had been spared. The shortage of medical staff at the infirmary, added to the other extra duties consequent upon the war, had a serious effect upon Dr. Maccormac's health, and, like others who remained in this country to minister to the wants of the sick, he had to perform the duties of those who have joined the Forces. It is to be hoped that he will long be spared to carry on the splendid work with which his name is so indissolubly associated with St. James's Infirmary, Wandsworth.

### MR. F. H. BENTHAM'S REVIEW OF POOR-LAW PROGRESS.

THOSE Poor-Law reformers who are tempted to pessimism by the contrast between their ideals and actuality may find some consolation in Mr. F. H. Bentham's review of the changes that have taken place in the Poor Law during the twenty-one years he has been connected with the Bradford Board of Guardians. In 1895 the Bradford Guardians had no proper Poor-Law hospital, no resident doctor, no dispenser, and only one trained nurse and four probationers. Such attention as the sick required was largely given by inmates. Now the Guardians possess a separate adult hospital, a children's hospital, a maternity hospital, a sanatorium for consumptives, and a colony for mental defectives, with resident doctors and a large nursing staff for each institution. Scattered homes for children have replaced the workhouse wards in which the children were formerly brought up, and institutional treatment is now given in preference to inadequate doles in the shape of out-relief. Mr. F. H. Bentham's statement was made on the occasion of his resignation of his membership of the Board. This resignation will be received with regret by all interested in Poor-Law matters. Mr. F. H. Bentham was a member of the Royal Commission on the Poor Laws, and one of the signatories to the Majority Report. The great changes which have been made in the Poor-Law administration of Bradford are largely due to his initiative and energy. The Poor-Law world loses by his resignation a devoted and unselfish worker.

### BUTTER V. MARGARINE.

BY twenty-four votes to twenty-three the Manchester Board of Guardians last week decided that its officers and the sick poor shall be provided with butter instead of margarine. Dr. Garrett urged that butter was more nutritious than margarine, which did not contain any albumen. Whatever may be the relative nutritive values of butter and margarine, Dr. Garrett's statement is incorrect. The amount of proteid material present in butter, as well as in margarine, is practically negligible from a dietetic point of view. The dietetic experts who condemn margarine rest their claim for the greater value of butter upon the alleged but unproved greater digestibility of animal as compared with vegetable fats, and upon the admitted presence in butter and absence from margarine of phosphorised fats and vitamines. The experience of medical men in charge of large institutions where the change from butter to margarine has been made shows conclusively that in the vast majority of cases the substitution can be made without any ill-effects if a good brand of margarine is used.

### THIS WEEK'S DRUG MARKET.

BUSINESS in the drug market has been fairly active during the week, and most of the price changes are in an upward direction. Cod-liver oil is in much the same position as it has been for some weeks; that is to say, the excessive prices quoted effectively keep buyers off the market. The advanced price of citric acid is well maintained, and citrates are much dearer. Sugar of milk continues to rise in price. Among synthetic drugs the most noteworthy change is in hexamine, which has again advanced in price in face of the continued demand. While there is no quotable change in the price of bromides the tendency is not quite so firm, and so far as can be foreseen any substantial advance is hardly likely to take place in the near future. Ipecacuanha being in more plentiful supply, the price is not quite so firmly maintained. Essence of lemon, oil of bergamot, and oil of orange are all slightly dearer.

### TO OUR READERS.

CONTRIBUTIONS are specially invited from any of our readers to these columns. They should deal with topical subjects and news. They must be authenticated for the information of the Editor only. The minimum payment if published is 5s. There is no hard-and-fast rule as to space, but notes of about twenty lines in length are preferred.

